# Molecular and histological effects of MR-guided pulsed focused ultrasound to the rat heart

**DOI:** 10.1186/s12967-017-1361-y

**Published:** 2017-12-13

**Authors:** Kee W. Jang, Tsang-Wei Tu, Matthew E. Nagle, Bobbi K. Lewis, Scott R. Burks, Joseph A. Frank

**Affiliations:** 1 Frank Laboratory, Radiology and Imaging Sciences, Clinical Center, National Institutes of Health, 10 Center Dr., Bethesda, MD 20892 USA; 2 National Institute of Biomedical Imaging and Bioengineering, National Institutes of Health, Bethesda, MD USA

**Keywords:** Focused ultrasound, Myocardium, Proteomics, Rat, Sterile inflammation, Lung trauma, Cardiac contusion

## Abstract

**Background:**

Image-guided high intensity focused ultrasound has been used as an extracorporeal cardiac pacing tool and to enhance homing of stem cells to targeted tissues. However, molecular changes in the myocardium after sonication have not been widely investigated. Magnetic-resonance (MR)-guided pulsed focused ultrasound (pFUS) was targeted to the rat myocardium over a range of pressures and the microenvironmental and histological effects were evaluated over time.

**Methods:**

Eight-to-ten-week-old Sprague–Dawley rats received T2-weighted MR images to target pFUS to the left ventricular and septum without cardiac or respiratory gating. Rats were sonicated through the thoracic wall at peak negative pressures (PNP) from 1 to 8 MPa at a center frequency of 1 MHz, 10 ms pulse duration and 1 Hz pulse repetition frequency for 100 pulses per focal target. Following pFUS, myocardium was harvested over 24 h and subjected to imaging, proteomic, and histological measurements.

**Results:**

pFUS to the myocardium increased expression of cytokines, chemokines, and trophic factors characterized by an initial increase in tumor necrosis factor (TNF)-α followed by increases in pro- and anti-inflammatory factors that returned to baseline by 24 h. Immediately after pFUS, there was a transient (< 1 h) increase in N-terminal pro b-type natriuretic peptide (NT-proBNP) without elevation of other cardiac injury markers. A relationship between PNP and expression of TNF-α and NT-proBNP was observed with significant changes (p < 0.05 ANOVA) ≥ 4 MPa compared to untreated controls. Contrast-enhanced ex vivo T1-weighted MRI revealed vascular leakage in sonicated myocardium that was accompanied by the presence of albumin upon immunohistochemistry. Histology revealed infiltration of neutrophils and macrophages without morphological myofibril changes in sonicated tissue accompanied by pulmonary hemorrhage at PNP > 4 MPa.

**Conclusions:**

MR-guided pFUS to myocardium induced transient proteomic and histological changes. The temporal proteomic changes in the myocardium indicate a short-lived sterile inflammatory response consistent with ischemia or contusion. Further study of myocardial function and strain is needed to determine if pFUS could be developed as an experimental model of cardiac injury and chest trauma.

**Electronic supplementary material:**

The online version of this article (10.1186/s12967-017-1361-y) contains supplementary material, which is available to authorized users.

## Background

Cardiovascular disease (CVD) represents a class of pathology that is considered one of the most serious public health threats. The prevalence is approximately 7 million people in the US and clinical manifestations include sinus node dysfunction, complete heart block, stroke, heart failure, cardiomyopathy and arrhythmia [1]. CVD can result in high blood pressure, myocardial infarction, stroke or sudden death if left untreated [2–4]. Numerous treatment options for CVD include lifestyle changes, pharmacological therapy, and surgery that have variable clinical outcomes depending on the severity of disease [5, 6]. The implantation of cardiac pacemakers, a common clinical option for treating arrhythmias, can result in improved cardiac function and quality of life [7]. However, this procedure is associated with some morbidity due to infection and the surgical procedure [8].

Image-guided high-intensity focused ultrasound (HIFU), using ultrasound (US) or magnetic resonance imaging (MRI) guidance, is a noninvasive technique that sonicates targeted regions in the body. It has been clinically approved to treat uterine fibroids, prostate cancer, and essential tremor [9–11]. Current clinical HIFU therapies apply continuous US to generate ablative temperatures within treatment volumes [12–14]. Alternatively, pulsed focused ultrasound (pFUS), that deposits primarily mechanical rather than thermal energy is experimental, but promising for several applications. It has been demonstrated to induce local molecular changes that increase tropism of systemically-infused stem cell therapies [15–19]. Mechanical forces from pFUS can be modulated by adjusting numerous US parameters such as pulse length, duty cycle, and peak negative pressure (PNP). The potential for pFUS as a noninvasive pacing tool has been superficially explored with promising results [20–25]. Previous reports have superficially examined proteomic and histological changes in ischemic myocardium following ultrasound-mediated microbubble destruction as a means to attract therapeutic stem cells [26–28], but systematic evaluation of pFUS effects on myocardial tissue and the possibility of coincident pulmonary contusion [29, 30] is virtually nonexistent.

This study therefore evaluated the mechanical non-thermal effects of MR-guided pFUS to the myocardium as a function of PNP to understand proteomic and histological changes over time. We observed that a transient sterile inflammatory response (SIR) in the treated myocardium that was associated with cardiac injury marker activation, inflammatory cell infiltration, and vascular leakage returning to baseline by 24 h post-pFUS.

## Methods

### Animals

All animal studies were approved by the Animal Care and Use Committee at the NIH Clinical Center, and experiments were performed in accordance with the National Research Council’s Guide for the Care and Use of Laboratory [31]. Female Sprague–Dawley rats (8–10 weeks of age, 230.2 ± 9.7 g; Charles River Laboratories, Wilmington, MA) were given free access to food and water. Hair on the chest was removed with depilatory cream prior to pFUS treatment. pFUS attenuation through the chest wall was determined by measuring relative transducer output with a hydrophone (HGL-1000, ONDA Corporation, Sunnyvale, CA) in degassed H_2_O. Measurements were recorded with and without excised rat chests (n = 3) placed in front of the hydrophone.

### MR-guided pFUS and MRI

**Fig. 1:**
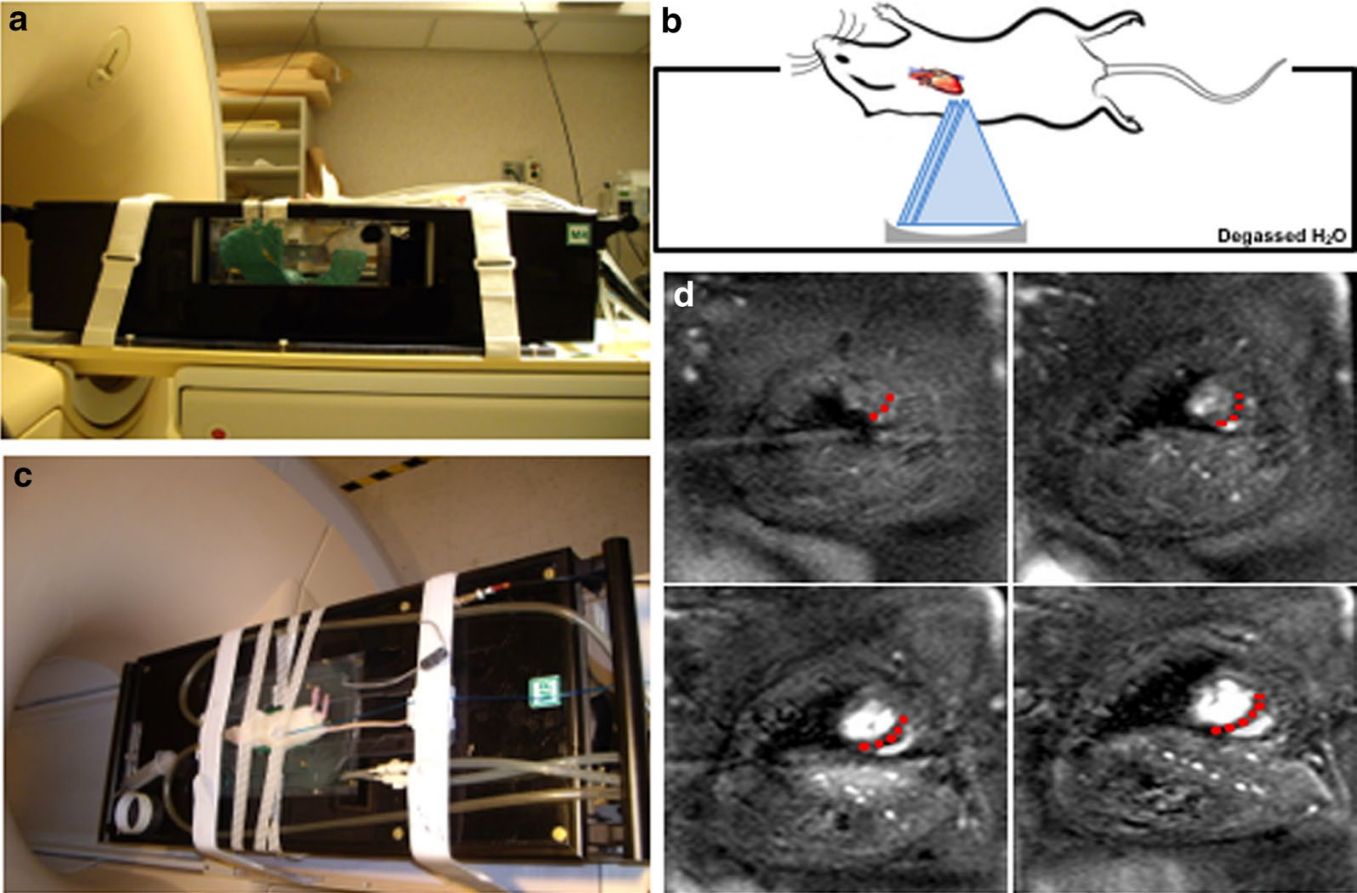
MR-guided pFUS. **a** Rats placed on a preclinical MR-compatible image-guided focused ultrasound system. **b** Illustration of pFUS treatment in the rat heart. **c** Left side of the rat chest was submerged in degassed H_2_O and a spherically focused ultrasound transducer was used to produce pFUS. **d** T2w coronal MR images were acquired at 1 mm slice thicknesses and used for pFUS guidance to target the left ventricular apex of the rat heart. Red dots represent pFUS-targeted areas

Rats (n = 85) were anesthetized with 1.5% isoflurane in 100% O_2_ and placed in the left lateral recumbent position on a preclinical MR-compatible image-guided focused ultrasound system (RK-100, FUS instruments, Ontario, Canada) with the left side of the chest submerged in degassed H_2_O maintained at 37 °C. Hearts were imaged without respiratory or cardiac gating using a radiofrequency receive coil on a 3T clinical MR scanner (Achieva, Philips Healthcare, USA) (Fig. 1a–c). Targeting MRI used multislice fast field echo (FFE) sequences with repetition time (TR) = 8.9 ms, echo time (TE) = 4.5 ms, flip angle (FA) = 45° and a slice thickness of 1 mm with an in-plane resolution of 0.14 × 0.14 mm^2^. pFUS was targeted toward the left ventricle (LV) and septum. The approximate LV length was 6.5 mm and the thickness of LV septum was 1.7 mm. The MRI slice thickness was similar to pFUS focal dimensions (diameter = 1.2 mm; length = 6 mm). pFUS was performed at a center frequency of 1 MHz using a transducer with a focal length of 6 cm and an electro-acoustic conversion efficiency of 75.9%. Sonications were performed at 1, 3, 4, 6, and 8 MPa PNP. The corresponding spatial averaged and temporal peak intensities (I_SATP_) were: (in W/cm^2^) 52 at 1 MPa, 472 at 3 MPa, 839 at 4 MPa, 1886 at 6 MPa, and 3355 at 8 MPa. Intensity values were confirmed by radiation force balance measurements. Using targeting images, approximately 20 foci in the LV and septum were treated with pFUS using the following US parameters: pulse length, 10 ms; duty cycle, < 1%; 100 pulses/site (Fig. 1d). Prior to pFUS, animals were given intravenous infusions of gadofosveset (200 µL/kg) (Lantheus Medical Imaging Inc., MA). Cardiac or respiratory gating was not used during sonication. At various times after pFUS, rats were euthanized and perfused with 4% paraformaldehyde (PFA) in 1× phosphate-buffered saline (PBS). Ex vivo post contrast T1-weighted (T1w) MRI was performed using a radiofrequency quadrature coil (Doty Scientific In., Columbia, SC) on a 7T MR microimaging system (Bruker Corp., Billerica, MA) with the following parameters: TR, 200 ms; TE, 8 ms; and resolution, 100 µm^3^. MR images were 3D reconstructed using Osirix software (Pixmeo, Geneva, Switzerland). The images were normalized to the mean signal intensity of each heart for a group-wise comparison. To calculate the fold changes of MR contrast following pFUS, the pixel intensity of T1w MR images in pFUS-targeted regions were normalized to an untreated region in the base region of the heart (n = 3/group).

To evaluate the pFUS-induced myocardial tissue permeability, Evans Blue dye (EBD) (50 mg/kg) (Sigma-Aldrich, St. Louis, MO) suspended in 1× sterilized PBS was administered intravenously prior to pFUS. Rats were euthanized and fixed as described above. EBD was detected in histological sections by fluorescence microscopy (λ_ex_ = 589 nm; λ_em_ = 615 nm) using a fluorescence slide scanner (Aperio FL, Leica Biosystem Inc., Buffalo Grove, IL).

### Molecular analyses

pFUS-treated myocardium (n = 5–10/time point) was harvested at 0.17, 1, 3, 6, 12, 18 and 24 h and was immediately frozen in liquid N_2_. Sham-treated rats (receiving pFUS with no power to the transducer) were also harvested and termed as 0 h. Samples were homogenized in cell lysis buffer containing a protease inhibitor cocktail (S8820-2TAB; Sigma-Aldrich, St. Louis, MO). The total protein content of homogenates was determined by bicinchoninic acid assay (23227; ThermoFisher Scientific, Waltham, MA). Homogenates (2 mg/mL total protein) were analyzed using a multi-plexed immuno assay for a rat cytokine/chemokine panel (RECYMAG65K27PMX; EMD Millipore, MA). The same homogenates were also analyzed by enzyme-linked immunosorbent assays (ELISA) for the following cardiac injury markers: heart-type fatty acid binding protein (H-FABP) (MBS725463; MyBioSource Inc., San Diego, CA), cardiac troponin I (cTnI) (LS-F23616; LSBio Inc., Seattle, WA), N-terminal pro b-type natriuretic peptide (NT-proBNP) (LS-F23593; LSBio Inc.), and tumor necrosis factor alpha (TNF-α) (AB46070; Abcam, Cambridge, MA) and heat shock protein 70 (HSP70) (AB133060; Abcam). All assays were performed according to manufacturers’ protocols and were read on a spectrophotometric plate reader (Spectra Max M5, Molecular Devices, Sunnyvale, CA).

### Histological analyses

For histological and fluorescent immunohistochemical (fIHC) analyses, rats were perfused with 4% PFA following euthanasia and equilibrated in 30% sucrose following overnight fixation in 4% PFA. For fIHC, frozen tissue sections (10 µm) were permeabilized with 0.1% Tween-20 in 1× PBS (TPBS) followed by antigen retrieval with Proteinase-K (Life Technologies, Frederick, MD). Sections were blocked with SuperBlock (ThermoFisher Scientific, Waltham, MA) and incubated with an appropriate primary antibody: FITC-conjugated sheep anti-albumin (10 µg/mL) (Abcam, Cambridge, MA), HIS48 (1:20 dilution) (Bio-Rad) or CD68 (10 µg/mL) (ab31630; Abcam) for 1 h at room temperature. The slides were then cover-slipped with a DAPI-containing mounting medium and imaged on a fluorescence slide.

To examine morphology, 10 µm sections of myocardium and lung tissue were stained with hematoxylin and eosin (H&E) and scanned with a bright-field slide scanner (Aperio SC2, Leica Biosystem Inc.). The fraction of pulmonary microhemorrhage in right lungs was analyzed within freehand-drawn ROIs over the total lung area using ImageJ (National Institutes of Health) (3 sections/animal, n = 3).

### Statistical analyses

All data are presented as mean ± standard deviation (SD) and data analyses were performed with Prism (version 7, GraphPad Software, Inc. La Jolla, CA). One-way analysis of variance (ANOVA) with Dunnett post hoc tests was used for multiple comparisons. The level of significance was set at *p* < 0.05 (*p < 0.05, **p < 0.01 and ***p < 0.001).

## Results

### MR-guided pFUS

Precise placement of the rats on the MR-guided pFUS system was necessary to orient the heart perpendicular to the ultrasonic transducer and minimize US exposure to the lungs (Fig. 1). There was approximately 40% attenuation of US intensity through the chest wall (Additional file 1: Figure S1). Treatment groups are identified by the PNP of the transducer output, however, intramyocardial PNP were estimated to be 0.6, 1.2, 1.8, 2.4, 3.6 and 4.8 MPa, for transducer outputs of 1, 2, 3, 4, 6, 8 MPa, respectively. Post-sonication MRI (FFE, TE = 5 ms, TR = 13 ms, FA = 45° and slice thickness was 2.5 mm with 3.5 s temporal resolution) revealed no injury to the right lung at PNP of 3 MPa; however, increased pulmonary signal intensity was observed at PNP ≥ 6 MPa (see Additional file 2: Figure S2).

### pFUS effect on molecular and cardiac injury markers

**Fig. 2:**
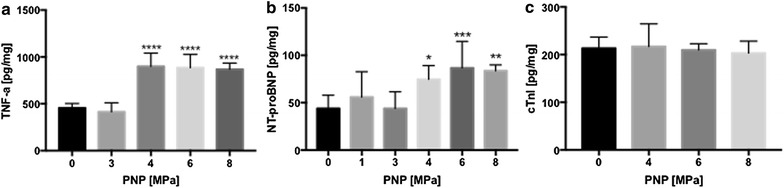
The effects of pFUS-PNP on cardiac injury marker expression. **a** TNF-α expression was significantly increased immediately following pFUS exposure at PNP greater than 4 MPa (n = 5). **b** NT-proBNP expression was significantly enhanced at PNP > 4 MPa immediately following pFUS. pFUS at 6 MPa showed the greatest increase in the NT-proBNP expression. **c** cTnI expression was not changed following pFUS at any PNPs (n = 4–5). Statistical significance was set at *p* < 0.05 following ANOVA

To evaluate the molecular effects of pFUS on myocardium, rats (n = 5/group) were sonicated at various PNP (i.e., 0 = sham, 1, 3, 4, 6, 8 MPa) and euthanized 4 h post-treatment. NT-proBNP, a marker of cardiac failure, was evaluated over the range of PNP studied. When the myocardium was sonicated with PNP > 3 MPa, NT-proBNP was significantly elevated compared to sham control (*p* < 0.05 ANOVA) (Fig. 2b). Elevations in NT-proBNP were observed up to 6 MPa. Based on this result, subsequent experiments were performed at 6 MPa. cTnI expression was not increased following pFUS at 6 MPa (Fig. 2c). HSP70 was not elevated by pFUS at any PNP tested (Additional file 3: Figure S3).

### Molecular responses to pFUS

**Fig. 3:**
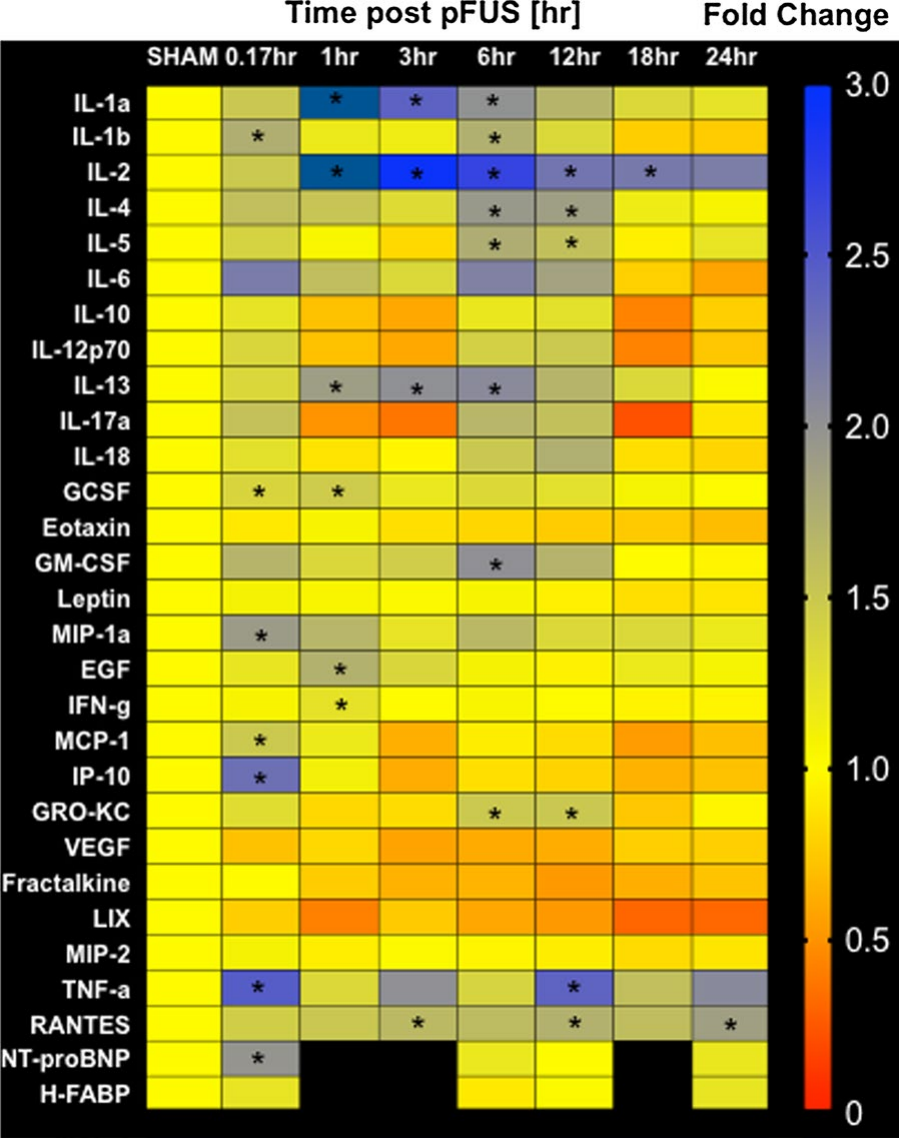
Proteomic response in pFUS-treated myocardium. Heat map depicting fold changes in CCTFs and cardiac markers over time following pFUS to myocardium. The levels of CCTFs and cardiac markers were quantified by ELISA and were normalized to sham control. Statistical significance was set at *p* < 0.05 following ANOVA

Within 10 min post-pFUS, TNF-α and interleukin (IL)-1β were significantly elevated (*p* < 0.05, ANOVA), as well as chemotactic factors granulocyte colony-stimulating factor (GCSF), macrophage inflammatory protein 1 alpha (MIP-1α), monocyte chemoattractant protein-1 (MCP-1), and interferon gamma-induced protein 10 (IP-10) (*p* < 0.05, ANOVA) (Fig. 3). Between 1 and 6 h post-pFUS, significant (*p* < 0.05, ANOVA) increases in expression of IL-1α, IL-2, IL-5, interferon (IFN)-γ, epidermal growth factor (EGF), granulocyte macrophage colony-stimulating factor (GM-CSF), growth-regulated oncogene keratinocyte chemoattractant (GRO-KC) and regulated on activation, normal T cell expressed and secreted (RANTES) were observed. During this time period, significant elevations in the anti-inflammatory cytokines IL-4 and 13 were also observed (Fig. 3). The pFUS-induced expression of cytokines, chemokines, and trophic factors (CCTF) returned to sham-control levels within 24 h (Fig. 3; Additional file 4: Figure S4 for the primary data). No increases in expression of IL-6, 10, 12, 17, vascular endothelial growth factor (VEGF) or MIP-2 following sonication at 6 MPa [16].

### Ex vivo MRI in sonicated myocardium

**Fig. 4:**
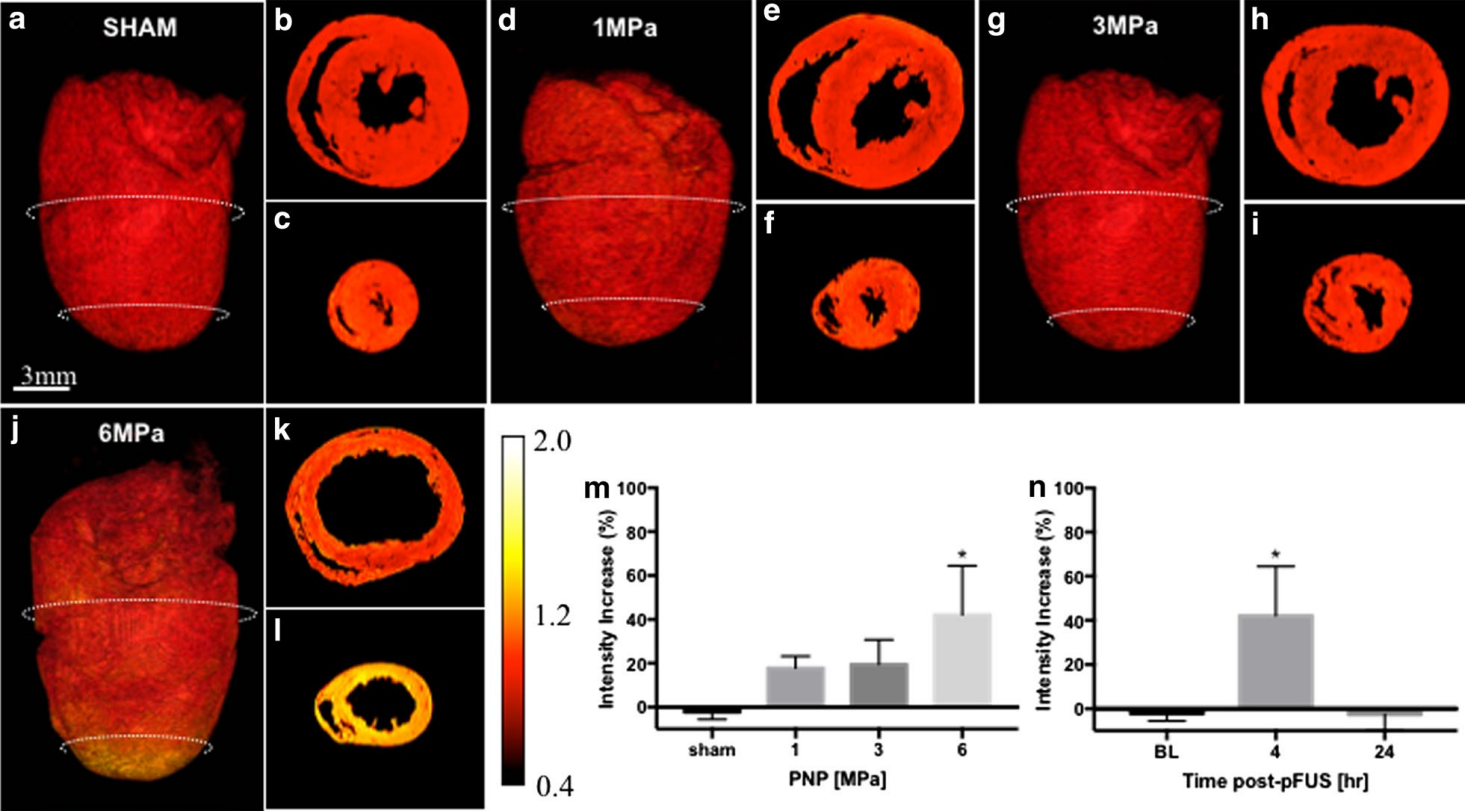
MRI changes in sonicated myocardium. Axial T1w MRI revealed little contrast enhancement following pFUS at 1 (**d**–**f**) and 3 MPa (**g**–**i**) compared to sham controls (**a**–**c**). However, pFUS at 6 MPa resulted in significant hyperintensities in the regions of pFUS (**j**–**l**). Contrast enhancement in pFUS targeted regions (**l**) was not observed in untreated myocardium (**k**). 3D reconstruction confirmed hyperintensities were distinctly located around the sonicated myocardium (**j**). Images were normalized to the mean signal intensity of each heart for a group-wise comparison. **m** Fold changes of in intensities were significant (*p* < 0.05) in hearts treated at 6 MPa. **n** Hyperintensities following pFUS at 6 MPa are not observed 24 h post-sonication

At 4 h post-pFUS, rats (n = 3) were perfused and hearts were harvested for ex vivo MRI studies. Each rat received an injection of gadolinium chelate prior to sonication in order to delineate changes in targeted areas in the myocardium by high resolution ex vivo MRI (Fig. 4). T1w images revealed contrast enhancement across the myocardial apex when compared to a section near the base (Fig. 4j–l) that was not present at PNPs ≤ 3 MPa (Fig. 4a–i). Compared to sham control, the enhancement was approximately 40% higher in myocardium treated at 6 MPa (Fig. 4m). Enhancement was not detectable by 24 h post-sonication (Fig. 4n). Ex vivo MRI revealed contrast enhancement located at the myocardial apex that was outside of the targeted area in the LV and septum.

### Histological analysis

**Fig. 5:**
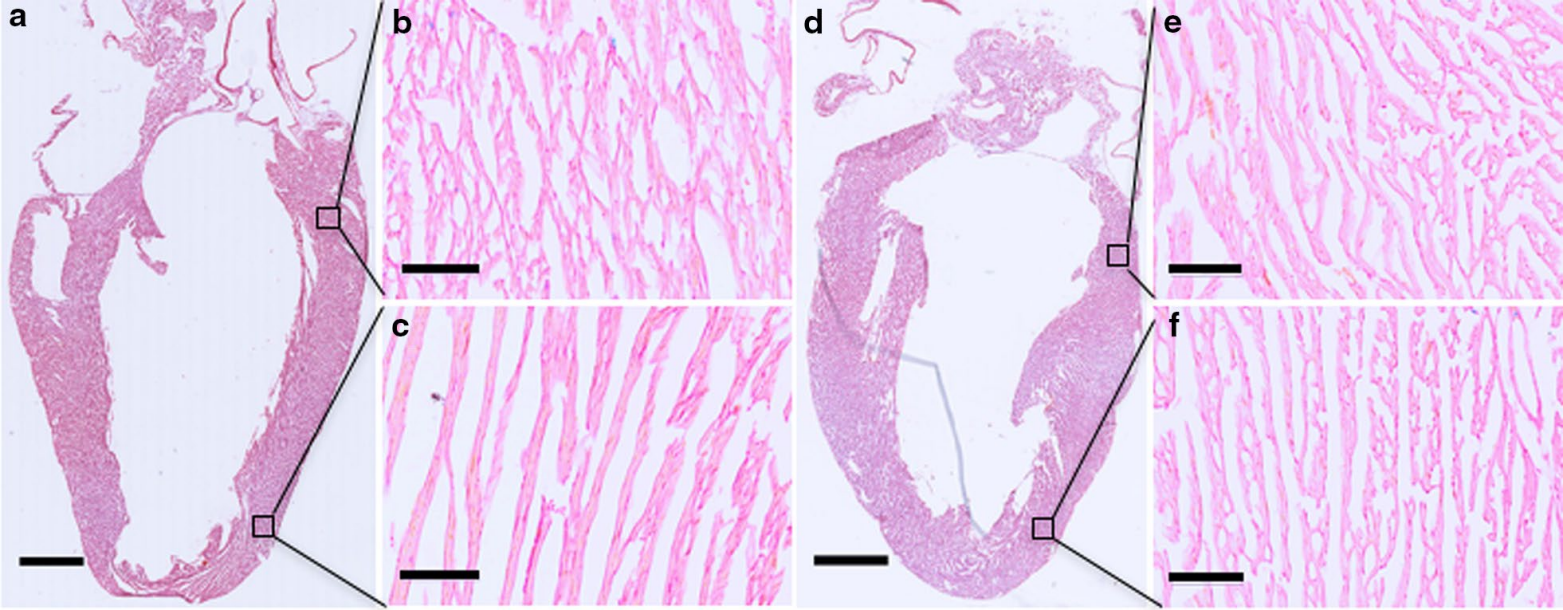
Histological evaluation of cardiac tissue following pFUS at 6 MPa. H&E staining revealed that no considerable differences between pFUS treated (**d**–**f**) and untreated regions (**a**–**c**). Low magnification scale bar = 2 mm; high magnification scale bar = 100 µm

**Fig. 6:**
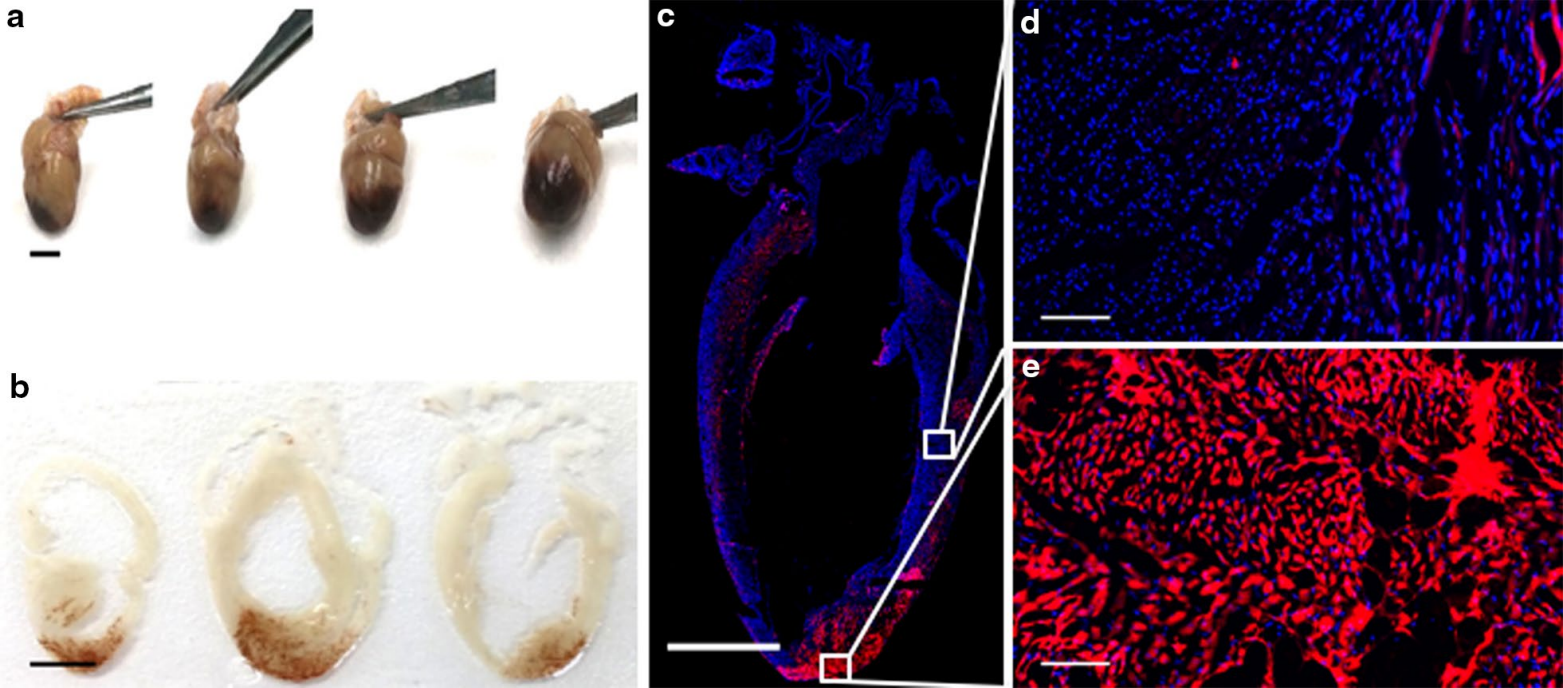
The effect of pFUS on EBD extravasation. **a** Gross examination shows significant EBD extravasation into the extracellular spaces, specifically in the region of pFUS treatment. **b** Macroscopic examination of tissue sections revealed that significant amount of EBD extravasated into sonicated myocardium. **c** fIHC analysis confirmed that the greatest amount of EBD was observed in sonicated region. Scale bar = 4 mm. **d**, **e** Show higher magnifications of pFUS untreated and treated regions. Scale bar = 100 µm. Blue/red represent DAPI/EBD respectively

H&E staining was performed on hearts harvested 4 and 24 h post-pFUS at 6 MPa and sham control. Neither red blood cell (RBC) extravasation (micro-hemorrhages) nor changes to histological architecture were observed following sonication (Fig. 5c, f). fIHC revealed the presence of albumin in extracellular spaces at the myocardial apex following sonication. Similarly, EBD fluorescence was detected in pFUS-treated myocardium (Fig. 6). The region of albumin (Additional file 5: Figure S5A–C) and EBD staining (Fig. 6) correlated to regions of contrast enhancement on the ex vivo MRI scans (Fig. 4). No considerable albumin staining was observed at 24 h post-pFUS treatment (Additional file 5: Figure S5D–F).

**Fig. 7:**
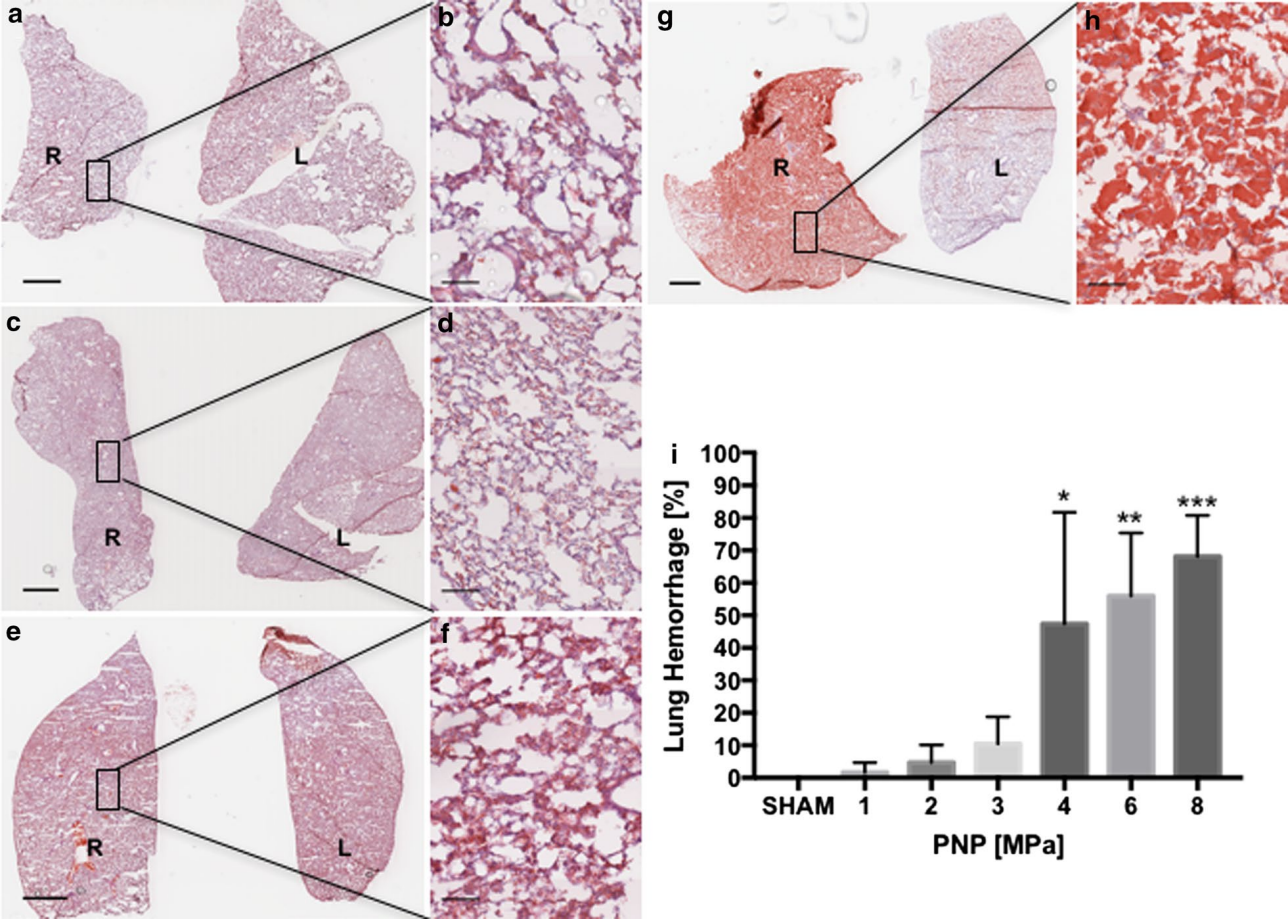
H&E staining of pFUS-treated myocardium. **a**, **b** Sham control revealed that little hemorrhage was observed. **c**, **d** pFUS exposure at 1 MPa showed no difference compared to sham. **e**, **f** pFUS exposure at 3 MPa produced little pulmonary hemorrhage. **g**, **h** pFUS exposure at 6 MPa caused extensive pulmonary hemorrhage. **i** Quantitation of pulmonary hemorrhage. **b**, **d**, **f**, and **h** are high magnification views of **a**, **c**, **e** and **g** respectively. L and R represent left and right lungs **a**, **c**, **e**, **g** scale bar = 2 mm. **b**, **d**, **f**, **h** Scale bar = 100 µm. Statistical significance was set at *p* < 0.05 based on ANOVA

The lungs are in the pFUS beam path and pulmonary exposure to pFUS is unavoidable. We examined lung sections from animals that received pFUS at 1, 3 and 6 MPa. H&E staining of the right lung revealed RBC extravasation in alveolar spaces with treatments > 4 MPa (Fig. 7). The percentage area of lung parenchyma with hemorrhage following pFUS was 47% at 4 MPa, 56% at 6 MPa, and 68% at 8 MPa (Fig. 7i). At lower pFUS PNP (i.e., 1 and 3 MPa) there was minimal evidence of pulmonary damage when compared to sham control (Fig. 7; Additional file 2: Figure S2).

**Fig. 8:**
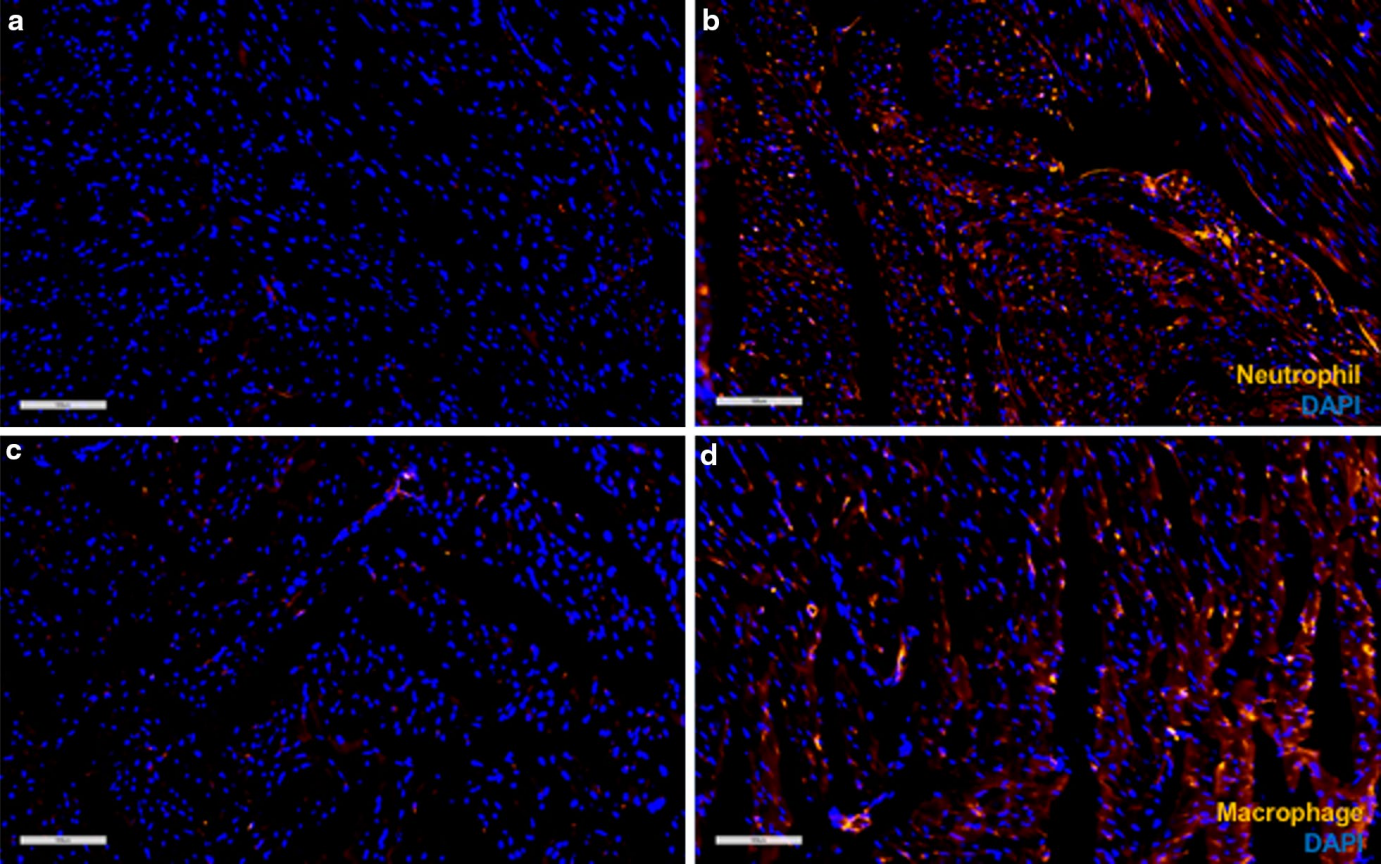
fIHC of macrophage and granulocyte infiltration into pFUS-targeted myocardium. **a**, **b** HIS48 staining showed more granulocytes in pFUS-targeted region compared to untreated regions. **c**, **d** CD68 staining showed more macrophages in pFUS-targeted region compared to untreated regions. Scale bar = 100 µm

To evaluate the innate immune response, fIHC analyses were performed at 24 and 48 h post-sonication. At 24 h, more macrophages (CD68^+^ cells) and granulocytes (HIS48^+^ cells) were observed in the myocardial apex compared to the untreated base of the heart (Fig. 8). Infiltrating macrophages and neutrophils became undetectable at 48 h post-pFUS (Additional file 6: Figure S6).

## Discussion

The major findings of this study were that non-gated MRI-guided pFUS targeted to the heart over a range of PNPs resulted in myocardial edema, and a transient pattern of molecular changes similar to a sterile inflammatory response (SIR). The SIR-like response was characterized by an early increase in TNF-α expression followed by upregulation of both pro- and anti-inflammatory CCTF that returned to baseline levels of expression by 24 h. We also observed a short-lived, but significant increase in the cardiac injury marker NT-proBNP within 10 min after sonication without increases in the cardiac injury markers cTnI or H-FABP. pFUS at ≥ 4 MPa revealed myocardial edema and pulmonary hemorrhage. The transient changes to the myocardium, as well as associated pulmonary hemorrhage are consistent with models of mild chest trauma and cardiac contusion.

The main purpose of this study was to examine the molecular and histological changes following pFUS at various PNP to the myocardium. It has recently been shown in preclinical models that image-guided FUS could be used as a noninvasive tool to pace and control heart rate [20–22]. These studies did not evaluate the effects of pFUS on cardiac biomarkers or subsequent molecular or histological changes within the myocardium or adjacent lung [29, 30]. To target pFUS to the myocardium, MRI was performed using 1-mm-thick sections that were sufficient to encompass the area of pFUS beam. Based on EBD extravasation in the myocardium following pFUS exposure, our results indicate that pFUS effects were confined primarily to the heart. We found this to be the best optimization of spatial resolution and signal for rapid imaging. This study began by performing preliminary experiments across a range of PNP to determine appropriate intensities for sonicating the rat myocardium. We have previously reported that the mechanical effects of pFUS in skeletal muscle and kidney induced rapid (within 30 min) increase in the expression of TNF-α compared to sham control when sonications were performed at 4 MPa [15–19]. Thus, we chose the same FUS parameters that we have used previously for the current study. Similar to the previous study, we observed significant increases in TNF-α within 15 min post pFUS starting at PNP of 4 MPa (not corrected for attenuation) (Fig. 2a). At PNP ≥ 4 MPa, significant increases in NT-proBNP, that is associated with myocardial stretch, was observed in the absence of other injury biomarkers. In order to maximize pFUS-induced CCTF and cell adhesion molecule (CAM) expression, we used 6 MPa. Previous reports using either pFUS or unfocused US to the myocardium combined infusions of microbubbles (MB) contrast agents provided limited evaluations of the CCTF and CAM response in the parenchyma. pFUS with MB infusion targeted to the peri-infarct regions in canine myocardium showed significant increases in IL-1β, VEGF, vascular cell adhesion molecule and stromal derived factor-1alpha [28]. Proteomic analysis following pFUS + MB infusion targeted to rat hearts resulted in significant increases in IL-1β, IL-4, IL-6, MCP-1, and TNF-α without increase in VEGF when compared to untreated control myocardium [27]. In a hamster model of chronic cardiomyopathy, the combination of US with MB infusion showed significant increases in mRNA expression and on histology of VEGF, basic fibroblastic growth factor and CAM along with an upregulation of TNF-α, IL-1β, and MCP-1 in the myocardium 3 days after sonication [26]. The latter study also infused bone marrow mononuclear cells following sonication and was able to demonstrate increased homing to the myocardium, along with improvements in myocardial perfusion, cardiac function, and decreased fibrosis [26]. Taken together these studies suggest that the molecular response by the myocardium to pulsed US + MB results in a pro-inflammatory response with increased expression of TNF-α and IL-1 that could be exploited to induce an enhanced homing and retention of infused stem cells to targeted tissues [16, 17, 19].

In the current study, there were significant elevations in pro-inflammatory CCTFs including TNF-α, IFN-g, IL-1α, IL-1β, IL-2, IP-10, MIP-1α, and MCP-1 within 1 h following sonication that would be consistent with cardiac injury. The rapid increase of both IL-1α and β post-pFUS indicate cardio-myofibril injury consistent with sterile inflammation presumably through an NF-kB pathway [32]. The increased expression of TNF-α and IL-1β and chemotactic factors (EGF, GM-CSF, RANTES, GRO-KC, GCSF, MIP-1α, MCP-1 and IP-10) following sonication has been associated with mechanical stretch and remodeling of myocardium [33–39]. We did observe transient early increase of NT-proBNP expression that has been associated with myocardial stretch and failure [40–42]; however, the biomarker did return to baseline levels during the study. There was also increased expression of EGF (a potent smooth muscle vasoconstrictor) that may have an impact on myocardial perfusion and changes in vascular permeability [36]. pFUS-induced expression of anti-inflammatory cytokines IL-4 and IL13 could limit a local SIR. The temporal molecular changes following pFUS would be consistent with a transient SIR that we have previously shown to occur following pFUS + MB exposure in the rat brain following the opening of the blood–brain barrier [43]. In the current study, we did not observe increased expression in VEGF and HSP70 following pFUS to the myocardium. These results were unexpected since cardiomyocytes express HSP70 under the various stress conditions and the lack of increase in VEGF was surprising since we have evidence of vascular leakage of albumin into the interstitial space [44–48]. Further investigation will be required to determine the myocardial response to pFUS from the basis of cellular response and stress (i.e., other types of HSP [49, 50]) and the lack of increased elevation of VEGF that is usually present following sonication to tissues [15–19].

There are few reports investigating cardiac injury marker responses, such as creatine kinase MB (CKMB), H-FABP, IL-6, TNF-a, cTnI and NT-proBNP from blood in cardiac contusion models [51–55]. Cardiac contusion models vary with respect to the distribution of the mechanical insults over the entire chest wall that may not cover the myocardium [56–59]. In the current study, MR-guided pFUS was used to target the myocardium resulting in mechanical insult to the heart and lungs. MRI examination of the rat myocardium following pFUS at > 6 MPa revealed gadolinium-chelate enhancement in the apex consistent with alteration in vascular integrity that is often observed in ischemia or contusion [60, 61]. The changes in vascular permeability with interstitial edema following pFUS has been observed in skeletal muscle [15, 62–64]. Histological examination of the sonicated myocardium staining did not reveal evidence of ischemia or microhemorrhages, However, immunofluorescent staining for albumin and EBD revealed extravasation into the interstitial space and an innate immune response with infiltration of neutrophils and macrophages was detected that cleared by 48 h.

The damage to the pulmonary microvasculature resulting in hemorrhage at PNP ≥ 4 MPa, and in combination with the CCTF gradient in the sonicated myocardium maybe responsible for the immune cells infiltration. There have been frequent reports that pulmonary damage is secondarily associated with blunt myocardial contusion due to the high kinetic energy transfer from the chest [59, 65–69]. Further investigation would be needed to understand the role of infiltrating macrophages and neutrophils in the myocardium following pFUS. Our observations suggest that amplification of the SIR by those cells could contribute to the remodeling of the transiently injured myocardium [70, 71].

There are several limitations of this study that need to be addressed. We were unable to synchronize the pFUS to cardiac and respiratory cycles due to technical limitations of the experiment that may have resulted in a greater amount of sonicated myocardium than treatment planning intended. Furthermore, the ultrasound injury to the lungs may have been avoided if it was possible to perform respiratory gating during sonication. Because of the position of the heart in the rat chest wall it was unlikely that pulmonary hemorrhage could have been avoided following sonication at any PNP. It is important to note that previous sonication studies targeting the heart did not comment on pulmonary injury presumably due to the lower PNP pressures coupled with the infusion of MB that resulted in acoustic cavitation in the myocardium [26–28]. We were also unable to acquire cardiac function studies to determine if pFUS altered various parameters that would be indicative of injury.

## Conclusions

The proteomic changes following pFUS to the rat heart was consistent with a transient SIR and innate immune response in the myocardium. We also observed a transient increase in NT-proBNP that would suggest myocardial stretching. The presence of interstitial edema upon contrast enhanced MRI and microscopy would suggest some compromise to vascular integrity that resolves over 24 h. The molecular and pathologic changes associated with pulmonary hemorrhages following pFUS suggest that using US treatment as an extracorporeal cardiac pacing tool could result in unwanted consequences requiring detailed treatment planning as well as cardiac and respiratory gating of the sonication. Further evaluation of cardiac function, such as left ventricular ejection fraction and myocardial strain, would be needed to determine if pFUS treatment could be used as a development of a model of cardiac injury and chest trauma.

## Additional files

**Additional file 1: Figure S1.** pFUS attenuation by the rat chest walls. pFUS intensity was attenuated approximately 40% by the rat chest wall.

**Additional file 2: Figure S2.** MRI of lungs pre- and post-pFUS. There were no changes in MR contrast in lungs of sham-treated rats (A and B) or rats treated at 3 MPa (C and D). (E and F) There were notable changes in signal intensity following pFUS exposure at 6 MPa. Scale bar = 10 mm.

**Additional file 3: Figure S3.** HSP70 expression following pFUS exposure. HSP70 expression was unchanged compared to sham controls following pFUS exposure at any PNP (n = 4–5).

**Additional file 4: Figure S4.** Time course of CCTF and cardiac markers. Quantitation of CCTF and cardiac markers after pFUS treatment. The y-axes represent picograms of cytokines per milligram of myocardium; the x-axes represent time [h] post-pFUS. Asterisks represent statistical significance of *p* < 0.05 based on ANOVA.

**Additional file 5: Figure S5.** Albumin staining. (A) fIHC revealed that greater amounts of albumin in pFUS-targeted regions after 4 h. Higher magnifications of (B) untreated and (C) treated regions. (D) Albumin staining 24 h post-pFUS showed no differences between pFUS-treated and untreated regions. Higher magnifications of (E) untreated and (F) treated regions. Blue/Green colors represent DAPI/Albumin respectively. Scale bar = 6 mm in A and D. Scale bar = 100 µm in B, C, E, and F.

**Additional file 6: Figure S6.** fIHC of macrophage and granulocyte infiltration into pFUS-targeted myocardium 48 h post-pFUS. (A and B) HIS48 staining showed no differences between treated and untreated regions. (C and D) CD68 staining showed no differences between treated and untreated regions. Scale bar = 100 µm.

## Abbreviations

ANOVA: one-way analysis of variance
CAM: cell adhesion molecule
CCTF: cytokines, chemokines, and trophic factors
CKMB: creatine kinase MB
cTnI: cardiac troponin I
CVD: cardiovascular disease
EBD: Evans blue dye
EGF: epidermal growth factor
ELISA: enzyme-linked immunosorbent assay
FA: flip angle
FFE: multislice fast field echo
fIHC: fluorescent immunohistochemical
GCSF: granulocyte colony-stimulating factor
GM-CSF: granulocyte macrophage colony-stimulating factor
GRO-KC: growth-regulated oncogene keratinocyte chemoattractant
H&E: hematoxylin and eosin
H-FABP: heart-type fatty acid binding protein
HIFU: high intensity focused ultrasound
HSP70: heat shock protein 70
IFN: interferon
IL: interleukin
IP-10: interferon gamma-induced protein 10
LV: left ventricle
MB: microbubbles
MCP-1: monocyte chemoattractant protein-1
MIP-1α: macrophage inflammatory protein 1 alpha
MP: megapascal
MRI: magnetic resonance imaging
NT-proBNP: N-terminal pro b-type natriuretic peptide
PBS: phosphate buffered saline
PFA: paraformaldehyde
pFUS: pulsed focused ultrasound
PNP: peak negative pressure
RANTES: regulated on activation, normal T cell expressed and secreted
RBC: red blood cell
ROI: region of interest
SD: standard deviation
SIR: sterile inflammatory response
TE: echo time
TNF- α: tumor necrosis factor alpha
TPBS: Tween-20 in 1× PBS
TR: repetition time
US: ultrasound
VEGF: vascular endothelial growth factor
