# Supplementary figures and images for: Molecular and histological effects of MR-guided pulsed focused ultrasound to the rat heart
Source: J Transl Med. 2017 Dec 13;15:252. doi: 10.1186/s12967-017-1361-y (PMC5729396; doi:10.1186/s12967-017-1361-y)

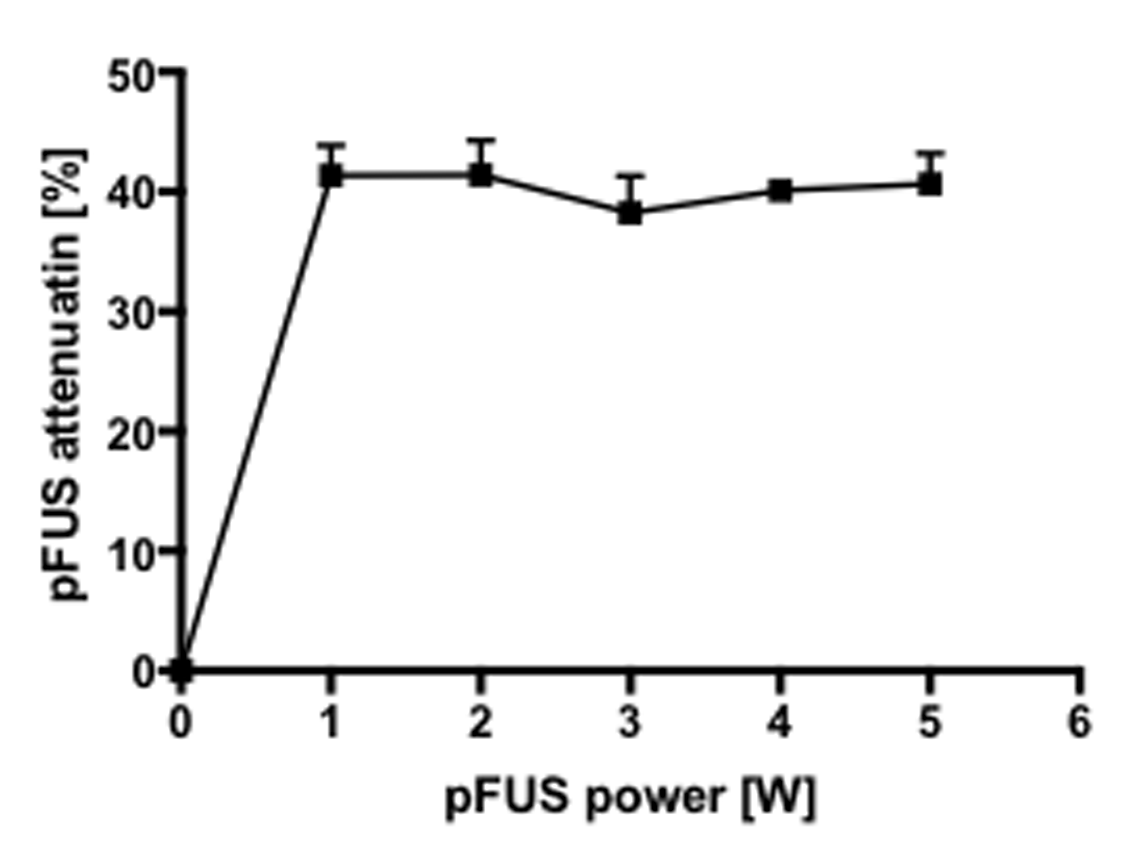

Supplement: Supplementary file 1 — Additional file 1: Figure S1. pFUS attenuation by the rat chest walls. pFUS intensity was attenuated approximately 40% by the rat chest wall. [file 12967_2017_1361_MOESM1_ESM.tif]

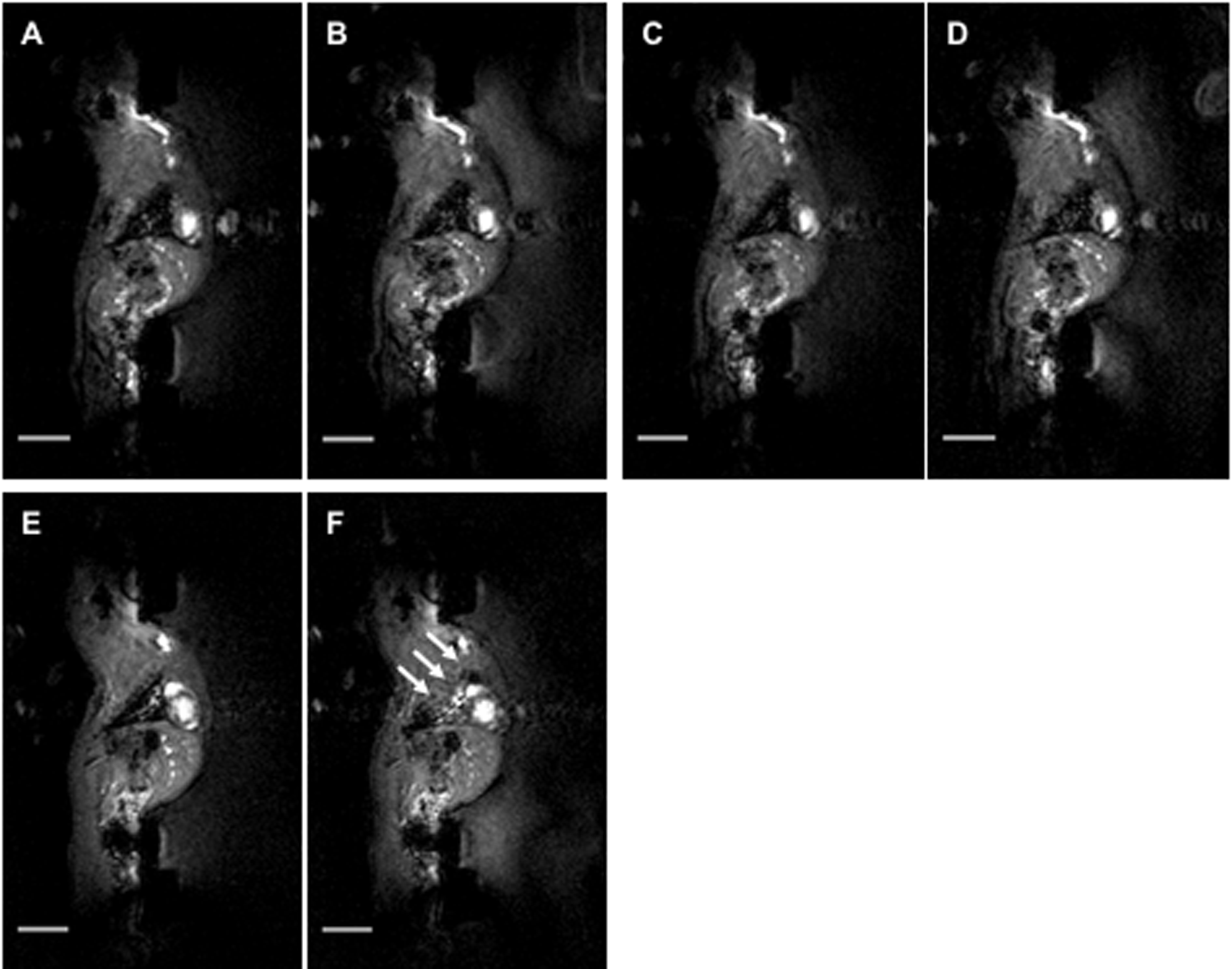

Supplement: Supplementary file 2 — Additional file 2: Figure S2. MRI of lungs pre- and post-pFUS. There were no changes in MR contrast in lungs of sham-treated rats (A and B) or rats treated at 3 MPa (C and D). (E and F) There were notable changes in signal intensity following pFUS exposure at 6 MPa. Scale bar = 10 mm. [file 12967_2017_1361_MOESM2_ESM.tif]

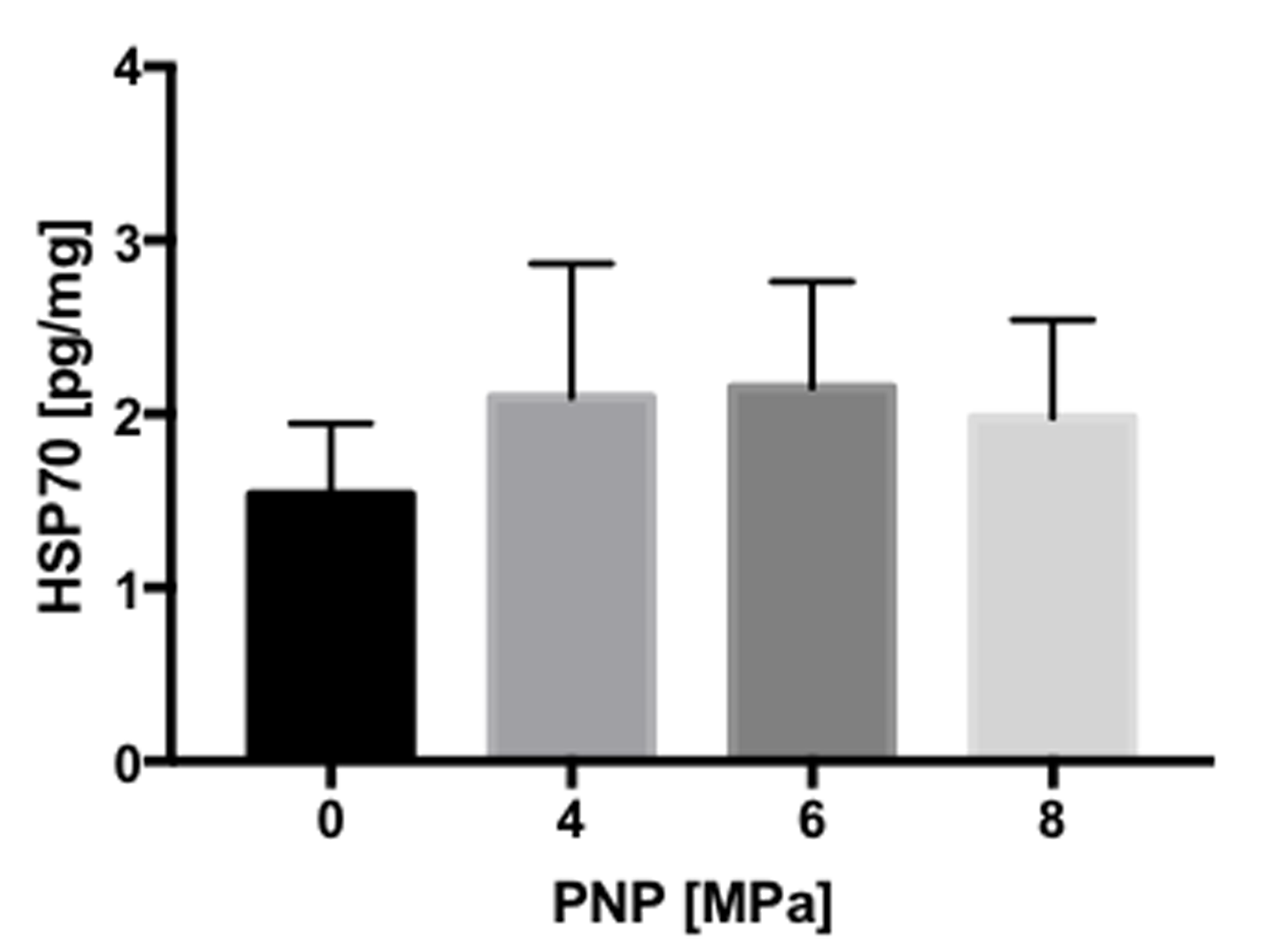

Supplement: Supplementary file 3 — Additional file 3: Figure S3. HSP70 expression following pFUS exposure. HSP70 expression was unchanged compared to sham controls following pFUS exposure at any PNP (n = 4–5). [file 12967_2017_1361_MOESM3_ESM.tif]

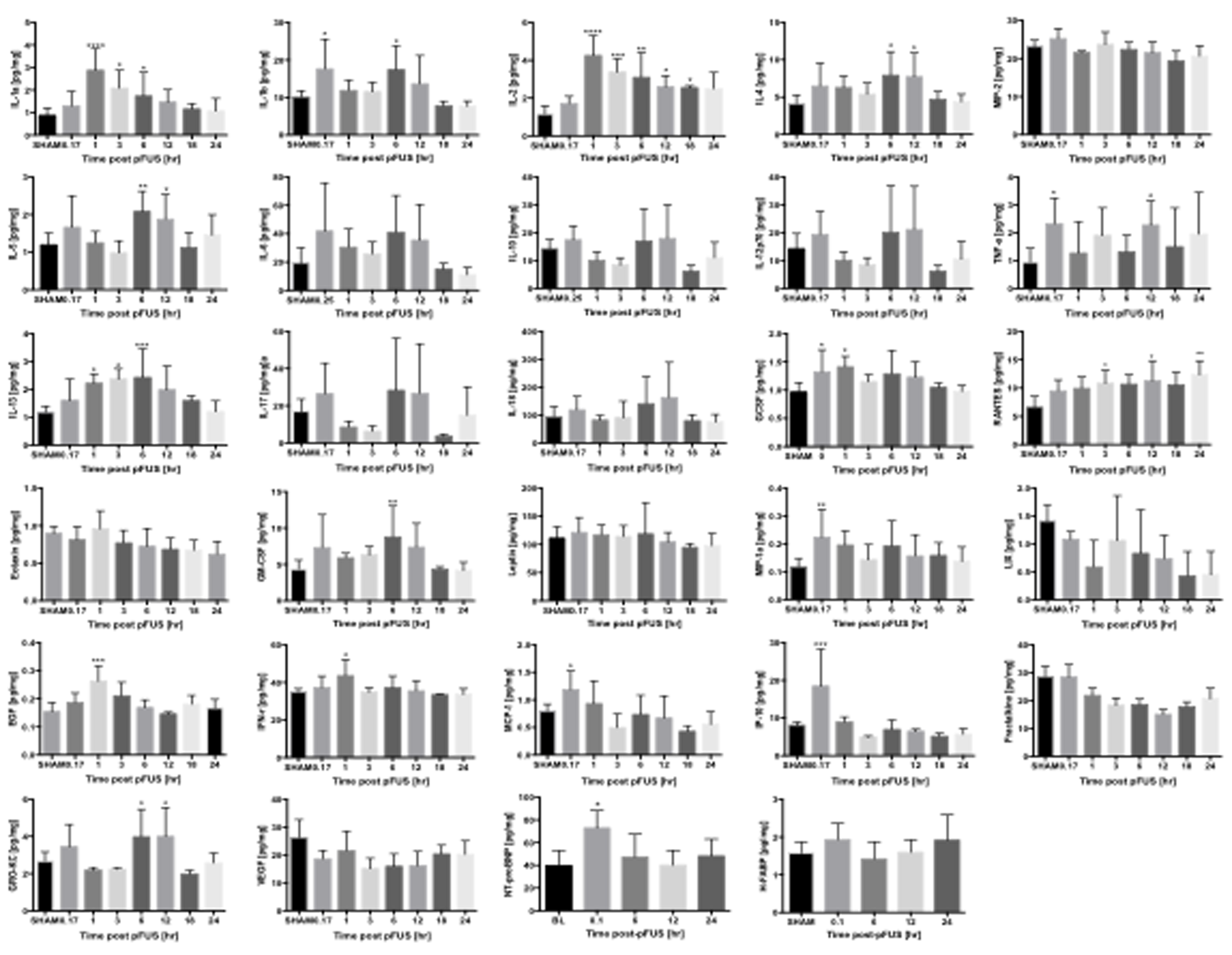

Supplement: Supplementary file 4 — Additional file 4: Figure S4. Time course of CCTF and cardiac markers. Quantitation of CCTF and cardiac markers after pFUS treatment. The y-axes represent picograms of cytokines per milligram of myocardium; the x-axes represent time [h] post-pFUS. Asterisks represent statistical significance of p < 0.05 based on ANOVA. [file 12967_2017_1361_MOESM4_ESM.tif]

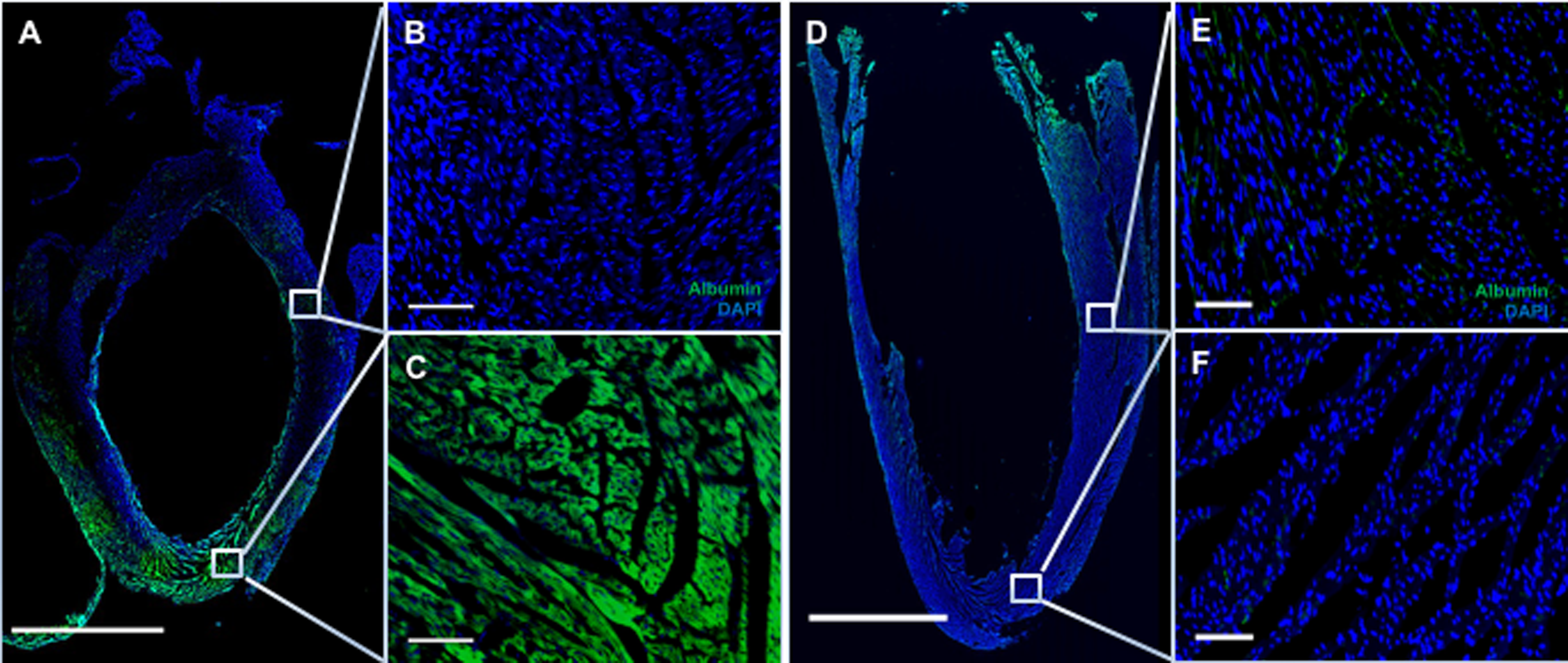

Supplement: Supplementary file 5 — Additional file 5: Figure S5. Albumin staining. (A) fIHC revealed that greater amounts of albumin in pFUS-targeted regions after 4 h. Higher magnifications of (B) untreated and (C) treated regions. (D) Albumin staining 24 h post-pFUS showed no differences between pFUS-treated and untreated regions. Higher magnifications of (E) untreated and (F) treated regions. Blue/Green colors represent DAPI/Albumin respectively. Scale bar = 6 mm in A and D. Scale bar = 100 µm in B, C, E, and F. [file 12967_2017_1361_MOESM5_ESM.tif]

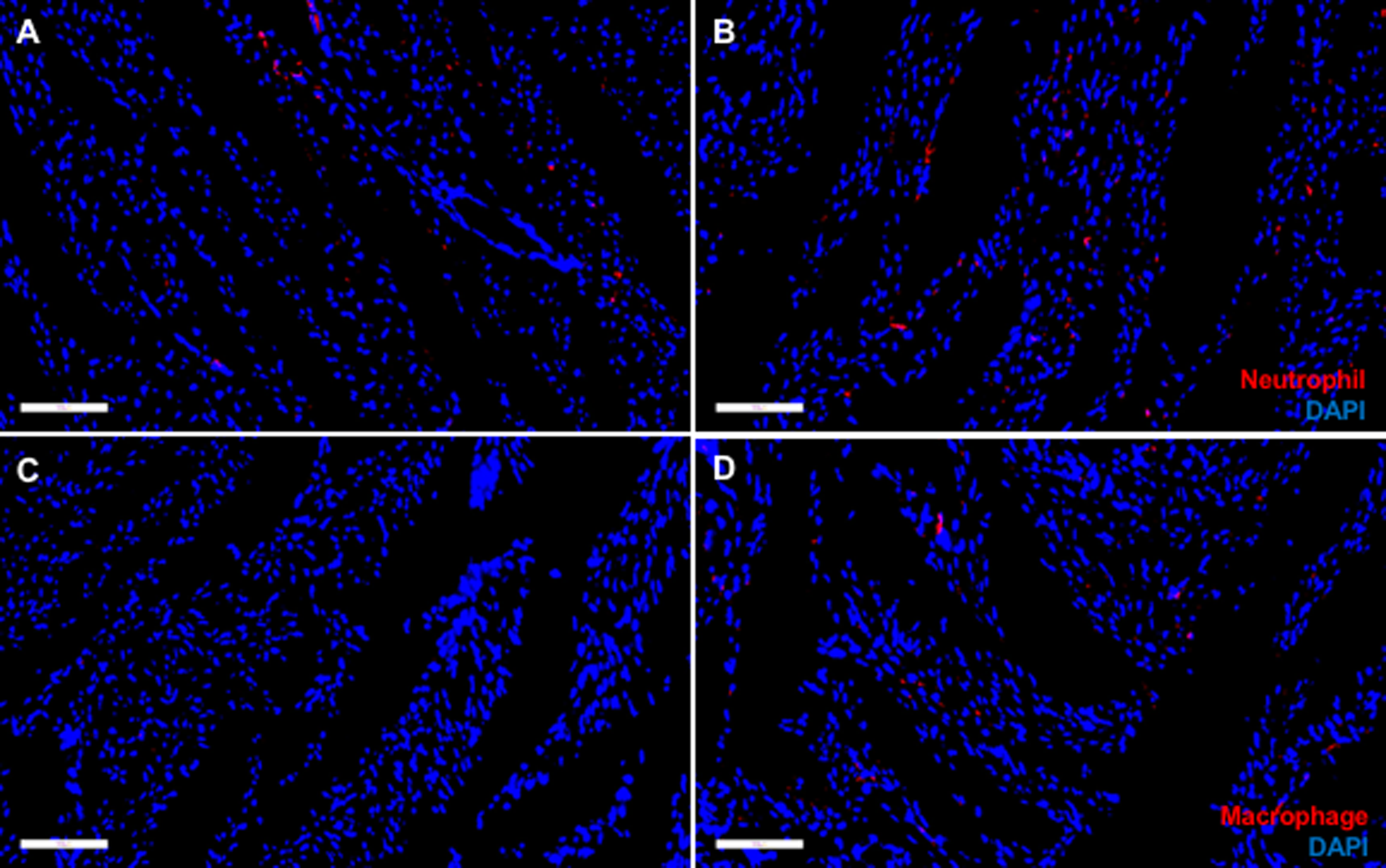

Supplement: Supplementary file 6 — Additional file 6: Figure S6. fIHC of macrophage and granulocyte infiltration into pFUS-targeted myocardium 48 h post-pFUS. (A and B) HIS48 staining showed no differences between treated and untreated regions. (C and D) CD68 staining showed no differences between treated and untreated regions. Scale bar = 100 µm. [file 12967_2017_1361_MOESM6_ESM.tif]
